# STAT6 Pathway Is Critical for the Induction and Function of Regulatory T Cells Induced by Mucosal B Cells

**DOI:** 10.3389/fimmu.2020.615868

**Published:** 2021-01-29

**Authors:** Kuan-Hua Chu, Szu-Yu Lin, Bor-Luen Chiang

**Affiliations:** ^1^Department of Pediatrics, National Taiwan University Hospital, Taipei, Taiwan; ^2^Graduate Institute of Immunology, National Taiwan University, Taipei, Taiwan; ^3^Graduate Institute of Clinical Medicine, National Taiwan University, Taipei, Taiwan; ^4^Allergy Center, National Taiwan University Hospital, Taipei, Taiwan

**Keywords:** STAT6, regulatory T cell, Peyer’s patch B cells, asthma, mucosal tolerance, LAG3

## Abstract

B cells could convert naïve T cells into regulatory T cells (so-called Treg-of-B cells) which have the ability to treat animal models of inflammatory diseases, including allergic asthma, collagen-induced arthritis and colitis; however, the mechanisms of Treg-of-B cell generation remain unclear. In this study, we investigated the role of STAT6 in the generation of Treg-of-B (P) cells, which Treg cells were generated by Peyer’s patch B cells (P stands for Peyer’s patch). CD4+CD25- T cells from wild type, STAT6 knockout and IL-4 knockout mice were cocultured with wild type Peyer’s patch B cells for Treg-of-B (P) cell generation. A murine asthmatic model was used to analyze the *in vivo* regulatory function of Treg-of-B (P) cells. The data demonstrated that STAT6 played a critical role in the generation of Treg-of-B (P) cells, which confirmed with STAT6-deficient T cells and the STAT6 inhibitor AS1517499. When STAT6 was lacking, Treg-of-B (P) cells exerted impaired suppressive ability with decreased LAG3 expression. Furthermore, Peyer’s patch B cells played an essential role in regulatory T cell generation. In the absence of Peyer’s patch B cells, T cells expressed decreased phosphorylated STAT6, which was followed by decreased LAG3 expression and impaired suppressive ability, suggesting that Peyer’s patch B cells provided the critical signal to activate STAT6 phosphorylation in T cells. Moreover, STAT6 deficient Treg-of-B (P) cells could not alleviate inflammation in an animal model of asthma *in vivo*. IL-4 was downstream of phosphorylated STAT6 and maintained Treg-of-B (P) cell survival with increased expression of Bcl-2 and Bcl_XL_. We reported a novel finding that the STAT6-LAG3 signaling axis is important for the induction and function of Treg-of-B (P) cells.

## Introduction

Asthma is a chronic inflammatory disease characterized by allergic airway inflammation with increased mucus production, lung epithelium remodeling (1) and airway hyperresponsiveness (AHR). In the past decade, many studies have tried to determine effective therapies to cure the disease. Recently, mucosal tolerance has been proven to be an effective mechanism for therapeutic approaches in a variety of immunological diseases (2). By definition, mucosal tolerance indicates that subjects do not respond to nonpathogenic antigens through mucosal routes. Regulatory T cells (Treg cells) play a critical role in maintaining mucosal tolerance. After oral or nasal administration of antigen, Treg cells develop and suppress subsequent immune responses (3–5). In recent years, many studies on Treg cells induced by B cells have been reported. This particular subset of Foxp3 negative Treg cells (Treg-of-B cells) exerts the ability to alleviate the severity of a variety of immune diseases, including asthma, colitis and rheumatoid arthritis (6–11).

Signal transducer and activator of transcription (STAT) family proteins are essential for T cell differentiation by transmitting cytokine signals to cells. The activation of STAT4, STAT3 and STAT5 is required for Th1, Th17, and Treg cell differentiation, respectively (12). STAT6 is considered to be the key of the IL-4 signaling pathway and a driver of Th2 cell differentiation (13). However, in the absence of IL-4R, Treg cells lose their regulatory function to suppress eosinophil infiltration in the lung after adoptive transfer into sensitized mice (14). Moreover, STAT6 plays a critical role in preventing allograft rejection (15). This finding implies that STAT6 might be important in regulating the function of Treg cells.

The role of LAG3 in immune tolerance has been extensively studied (16–19). LAG3 participates in the function of Treg cells both *in vitro* and *in vivo*. In tumor-bearing mice, LAG3 synergistically cooperated with PD-1 and contributed to tumor escape from immunosurveillance (20). Ectopic expression of LAG3 in CD4 T cells could confer these T cells with a regulatory function. Our studies also demonstrated that LAG3 participates in the suppressive function of Treg-of-B cells (8, 21). The use of an anti-LAG3 antibody to block LAG3 abrogated the inhibitory effect of Treg-of-B (P) cells on responder T cell proliferation. However, the mechanisms by which B cells induce LAG3 expression on T cells remain unclear.

Cytokines could modulate lymphoid cell growth, differentiation, survival and function. Previous studies reported that common gamma chain cytokines, including T cells, B cells, monocytes and Treg cells, play a key role in maintaining cell proliferation and survival, (22, 23). IL-4, which transduces signals through the common gamma chain receptor, could regulate Treg cell function. Deprivation of IL-4 downregulated the suppressive ability of Foxp3+ CD4+ Treg cells (24). However, some studies demonstrated that IL-4 inhibited Treg cell regulatory function by interfering with Foxp3 stability (25, 26). Given that IL-4 is the activator and downstream of STAT6 and the opposite role of IL-4 in Treg cell function, we aimed to determine whether IL-4 participates in Treg-of-B (P) cell generation.

In this study, we demonstrated a novel finding that Peyer’s patch B cells induce Treg-of-B (P) cell generation by phosphorylating STAT6 in T cells, leading to LAG3 expression. IL-4, which is activated by phosphorylated STAT6, did not affect Treg-of-B (P) cell generation but participated in maintaining Treg-of-B (P) cell viability. Moreover, STAT6-deficient Treg-of-B (P) cells exhibited an impaired ability to modulate immune responses in asthmatic mice. In conclusion, the STAT6-related pathway plays a critical role in Treg-of-B (P) cell generation.

## Materials and Methods

### Animals

STAT6-deficient (STAT6KO) BALB/c background mice were obtained from Jackson Laboratory (Bar Harbor, ME). IL4-deficient (IL4KO) C57BL/6 background mice were kindly provided by Prof. Shen’s laboratory in Chang Gung University. STAT6KO and IL4KO mice were bred in the National Laboratory Animal Center (Taiwan). C57BL/6 mice, BALB/c mice and OVA-TCR transgenic (DO11.10) mice aged 6–8 weeks were obtained and maintained in the National Laboratory Animal Center. The Institutional Animal Care and Use Committee (IACUC) of the College of Medicine at National Taiwan University approved the animal study protocol.

### Preparation of Treg-of-B Cells

The protocol for Treg-of-B cell generation and the characteristics of these cells are as previously described (6). Briefly, wild type, STAT6KO or IL4KO naïve CD4 T cells from spleens were enriched by negative isolation *via* immunomagnetic depletion (EasySep, STEMCELL Technology, Canada) to purity of more than 90%. Separation of Peyer’s patch B cells resulted in purity between 90% and 95% by B220 expression *via* immunomagnetic positive selection (IMag, BD Pharmingen). CD4+CD25- T cells were cultured with B cells (B:T=1:1) in presence of soluble anti-CD3 and anti-CD28 0.5 µg/ml in culture medium (RPMI-1640 supplemented with 5% FBS, 25 mM HEPES, 4 mM L-Gln, 100 U/ml penicillin, 100 μg/ml streptomycin, and 0.25 μg/ml amphotericin) for 3 days.

To determine the role of different STAT phosphorylation in Treg-of-B (P) cell generation, STAT inhibitors were added in Treg-of-B (P) cell generation step. STAT inhibitors: Fludarabine (Fludara, 50 µM, STAT1 inhibitor), Stattic (10 µM, STAT3 inhibitor) and SH4-54 (10 µM, STAT3 and STAT5 inhibitor), were purchased from Targetmol (Boston, MA), and AS1517499 (AS, 50 nM, STAT6 inhibitor) was purchased from Axon (Groningen, The Netherlands). Neutralizing antibody against IL-4 (10 µg/ml, BD Pharmingen) was used in the Treg-of-B (P) cell preparation step to clarify the role of IL-4 in Treg-of-B (P) cell generation.

In LAG3 induction, T cells were cultured with anti-CD3 (0.5 µg/ml) and anti-CD28 (0.5 µg/ml) in presence or absence of B cells for 3 days and then used for LAG3 detection. For the fully activation, plate-immobilized anti-CD3 antibody was applied for T cells cultured without B cells, whereas the soluble anti-CD3 antibody was used in B-T culture.

For detection of the cytokine production by Treg-of-B (P) cells, after three days B/T cocultured, Peyer’s patch B cells were depleted and Treg-of-B (P) cells were harvested and restimulated by plate-immobilized anti-CD3 and CD28 antibodies 1 µg/ml for 48 h. Supernatants were collected for cytokine assay by ELISA.

### Suppressive Function

The assessment of suppressive function, which means the ability of Treg cells to inhibit responder T cell proliferation, has been described previously (21). After three-day Treg-of-B (P) cell generation, Treg-of-B (P) cells were harvested and cultured with CD25^-^CD4^+^ T cells (as responder T cells) and splenocytes, treated with 25 µg/ml mitomycin c in 37°C for 30 min as antigen-presenting cells, in the presence of anti-CD3 and anti-CD28 1 µg/ml for 96 h. Proliferative response was measured by the addition of 1 µCi ^3^H-thymidine into the culture for the last 16 h. Thymidine uptake was determined using a β-counter (Packard Instrument Co., Meriden, CT, USA) and expressed as cpm (counts per minute).

### Fluorescence-Activated Cell Sorting (FACS) Analysis

For cell surface marker staining, monoclonal antibody (mAb) against PD-1, CTLA4, GITR, TNFRII and LAG3 were purchased from BD Pharmingen; mAb against OX40 was purchased from Biolegend (San Diego, CA, USA); and Ab against ICOS was purchased from eBioscience (San Diego, CA, USA). For apoptotic associated protein, Bcl-2 (BD Pharmingen), Bcl_XL_ and Bax (Santa Cruz, Texas, USA). Phosphorylated STAT (pSTAT) was stained with mAbs against pSTAT1, pSTAT3, pSTAT4, pSTAT5, pSTAT6 (BD Phosflow) and pSTAT2 (Merck Millipore) followed by an intracellular staining protocol. For determination of apoptosis, cells were stained with Annexin V (BD Pharmingen) and Propidium Iodide (PI, Sigma) followed by an apoptotic staining protocol. Cells were analyzed on a FACSCalibur and FACSLyric (BD Biosystems, Franklin Lakes, NJ, USA). Data were analyzed with FlowJo.

### Apoptosis Assay

Naïve T cells were cultured with Peyer’s patch B cells in presence of soluble anti-CD3 and anti-CD28 1 µg/ml for 24 to 72 h. Cells were stained with Bcl-2, Bcl_XL_, Bax, Annexin V and PI followed by manufacturer’s instructions.

### Real-Time PCR

Total RNA was isolated from wild type and IL4KO Treg-of-B cells using Trizol reagent (Invitrogen, Life Technology) and thereafter reverse-transcribed into cDNA using random hexamers (SMART RT-PCR kit, *BD* Biosciences *Clontech*). Gene expression of Bcl-2, Bcl_XL_ and Bax were determined in triplicates by quantitative real-time PCR using SYBR Gene Expression Assays according to the manufacturer’s protocol on an ABI 7500Fast (Applied Biosystems, Life Technology, CA, USA). Amplification of the endogenous control GAPDH was performed in order to standardize the amount of sample cDNA added. Real-time PCR primer: GAPDH forward: 5’-GATGGGTGTGAACCACGAGA-3’, reverse: 5’-AGATCCACGACGG ACACAT-3’. Bcl-2 forward: 5’-TGAGTACCTGAACCGGCATCT-3’, reverse: 5’-GCATCCCAGCCTCCGTTAT-3’. Bcl_X_ forward: 5’-ACCACCTAGAGCCTTGG ATCC-3’, reverse: 5’-TCTCGGCTGCTGCATTGTT-3’.

### Cytokine Detection by ELISA

IL-4, 5, 10, and eotaxin production were analyzed by an ELISA kit (R&D, Minneapolis, MN, USA) according to the manufacturer’s instructions.

### Adoptive Transfer of Treg-of-B Cells for the Alleviation of OVA-Induced Allergic Airway Inflammation

OVA-induced airway inflammation was established as described in the previous study (6). Six to 8-week-old BALB/c mice were sensitized by intraperitoneal injections of 50 μg OVA emulsified in 4 mg of alum on day 0, and 25 μg OVA mixed with 4 mg of alum on days 14 and 21. On days 36–38, mice were challenged with OVA 100 μg/mouse (in total volume 40 μl) by intranasal administration. On day 39, airway hyperresponsiveness was measured and mice were killed on day 40. Wild-type Treg-of-B (P) cells and STAT6-deficient Treg-of-B cells were prepared (2.5 × 10^6^ cells per mouse) and injected intravascularly into mice on day 1 and 14. Asthmatic control mice were injected in a similar manner with PBS. The naive group received challenge but without prior sensitization (**Figure 4A**). Airway hyperresponsiveness (AHR) was measured, and the mice were sacrificed after challenge. OVA-specific antibodies, airway hyperresponsiveness, and bronchial alveolar lavage fluid (BALF) were analyzed to evaluate the effects of Treg-of-B cells on airway inflammation.

Infiltrated eosinophils in BALF were counted and classified with the expression of MHC-II and CCR3 (27). Briefly, granulocytes were recognized as nonautofluorescent highly granular (SSChi) cells, and within this gate eosinophils were defined as cells expressing the CCR3, and very low to undetectable expression of MHC-II. Neutrophils had a similar scatter profile to eosinophils, but lacked CCR3 expression.

The airway responsiveness to aerosolized methacholine (MCh) (Sigma, St. Louis, MO, USA) was measured as described previously. The mice were placed into the main chamber (Buxco Electronics. Inc., Sharon, CT, USA) and challenged with aerosolized 0·9% normal saline, accompanied by increasing doses of MCh (6·25–50 mg/ml). The Penh [enhanced pause = pause × (peak expiratory box flow/peak inspiratory box flow)] values were determined. The Penh value was expressed as a relative increase ratio in response to PBS challenge.

### Histopathological Study

After lavage, the lungs were immediately removed, fixed in 10% buffered formalin, and embedded in paraffin. Sections (5 µm thick) were stained with hematoxylin-eosin (H&E) and examined by light microscopy for histological changes. To grade the extent of lung inflammation, semiquatitative scoring system was used as previously described (28). Briefly, infiltrated cell counts were performed blind based on five point grading system for the following features: 0, normal; 1, few cells; 2, a ring of inflammatory cells 1 cell layer deep; 3, a ring of inflammatory cells 2–4 cells deep; 4, a ring of inflammatory cells of >4 cells deep.

### Statistical Analysis

The results were expressed as the mean ± standard error of the mean (SEM). Statistical analyses were performed using GraphPad Prism VII software (GraphPad Software, La Jolla, CA). The Mann–Whitney U test was used for determination of the significance of differences between two groups. A *p* < 0.05 was considered statistically significant.

## Results

### STAT6 Was the Key Factor for LAG3 Expression and Treg-of-B (P) Cell Generation

**Figure 1:**
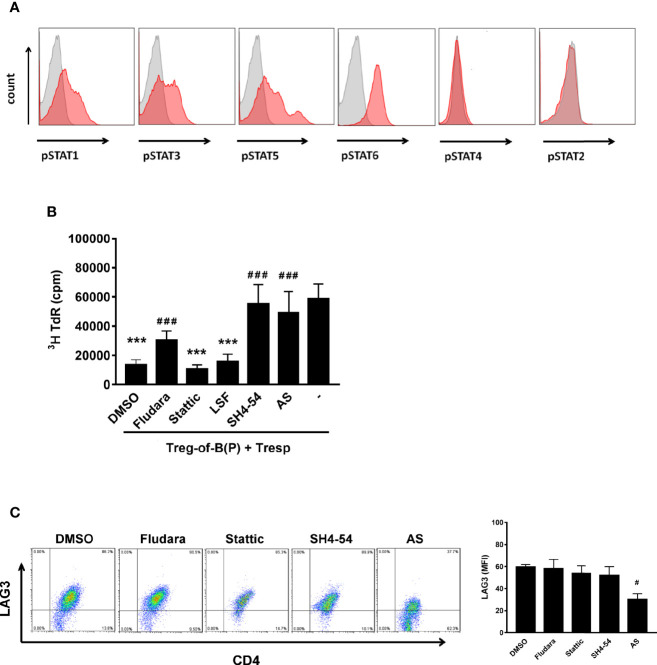
Phosphorylated STAT6 was the major STAT to regulate LAG3 expression and Treg-of-B (P) cell generation. To investigate whether phosphorylated STATs participated in Treg-of-B (P) cell generation, STATs phosphorylation was evaluated by FACS analysis. After culturing with Peyer’s patch B cells in presence of anti-CD3 and anti-CD28 antibodies for three day, CD4+CD25- T cells become Treg-of-B (P) cells with phosphorylated STAT1, 3, 5, and 6 **(A)**. To discover which STAT was the most important factor for Treg-of-B (P) generation, different STAT inhibitors were applied in the step of Treg-of-B (P) generation. The suppressive function (described in Materials and Methods section) and LAG3 expression by Treg-of-B (P) cells were used as the readout **(B**, **C)**. SH4-54 and AS group showed abolished suppressive ability indicated that phosphorylated STAT5 and STAT6 mediated the Treg-of-B (P) cell induction **(B)**. Moreover, STAT6 regulated the expression of LAG3 by Treg-of-B (P) cells **(C)**. Different STAT inhibitor treated Treg-of-B (P) cells were labeled as following: STAT1: Fludara, 50µM; STAT3: Stattic. 10µM; STAT3/5: SH4-54, 10µM; STAT6: AS, AS1517499, 50nM. Histogram: gray, isotype control; red, phosphorylated STAT. Responder T (Tresp) cell only group was labeled as “-”. Data are representative of three different experiments. Results are expressed as the mean ± SEM. ***p < 0.005, compared with responder T cell only group. ^#^*p* < 0.05, ^###^*p* < 0.005, compared with DMSO treated group.

Given that STAT phosphorylation affects T cell differentiation (12, 29), we assessed how phosphorylated STAT modulated Treg-of-B (P) cell function. Fluorescence-activated cell sorting (FACS) data showed that, in contrast to STAT2 and STAT4, STAT1, STAT3, STAT5, and STAT6 were phosphorylated in T cells cultured with Peyer’s patch B cells (**Figure 1A**). To determine which STAT was important for Treg-of-B (P) cell induction, we evaluated the suppressive function of different Treg-of-B (P) cells induced by Peyer’s patch B cells in the presence of different STAT inhibitors, including Fludarabine (Fludara, STAT1 inhibitor), Stattic (STAT3 inhibitor), SH4-54 (STAT3 and STAT5 inhibitor) and AS1517499 (AS, STAT6 inhibitor). The results showed that STAT5 and STAT6 were important for Treg-of-B (P) cell generation. Given the lack of STAT5 and STAT6 phosphorylation, Treg-of-B (P) cells could not be induced to suppress responder T cell proliferation (**Figure 1B**). In contrast, STAT3 had no effect, and STAT1 was partially involved in Treg-of-B cell induction. Furthermore, we determined which STAT controlled the expression of LAG3 by Treg-of-B (P) cells because LAG3 mediates the suppressive function of Treg-of-B (P) cells (8, 21). LAG3 expression was significantly decreased by abrogating phosphorylated STAT6, but not other STATs (**Figure 1C**), suggesting that STAT6 could regulate the expression of LAG3, which might lead to the impaired generation of Treg-of-B (P) cells.

**Figure 2:**
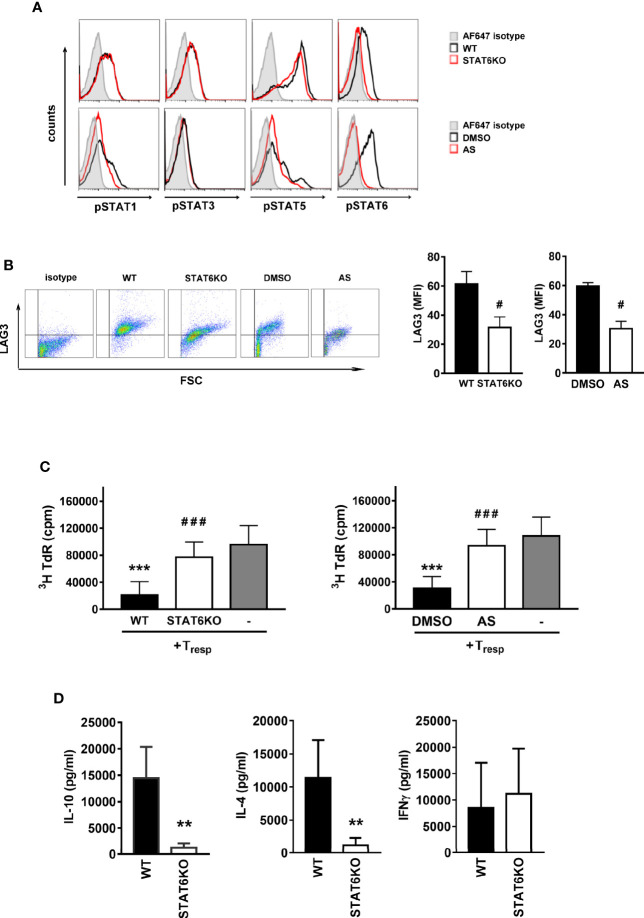
STAT6 deficient Treg-of-B (P) cells expressed lower LAG3 level and suppressive ability. In order to demonstrate the important role of STAT6 for Treg-of-B (P) cell generation, genetic deletion of STAT6 or the chemical inhibition of STAT6 phosphorylation were carried out. Wild type (WT) and STAT6 knockout (STAT6KO) CD25- CD4+ T cells were cultured with Peyer’s patch B cells or wild type CD25-CD4+ T cell cultured with Peyer’s patch B cell in presence of DMSO (control group) or AS (STAT6 inhibition group) for 3 days. After three-day cultured, Treg-of-B (P) cells were harvested and applied for FACS analysis, including phosphorylated STAT1, 3, 5, and 6 **(A)**, LAG3 expression **(B)** and suppressive function test **(C)**. **(D)** The cytokine levels of IL-4, IL-10, and IFNγ secreted by WT and STAT6KO Treg-of-B (P) cells. After 3-day cocultured, Peyer’s patch B cells were depleted, Treg-of-B (P) cells were harvested and re-stimulated with plate-bound anti-CD3 plus soluble CD28 1 µg/ml. after 48 h stimulation, the supernatant was collected for cytokine evaluation by ELISA. Results are expressed as the mean ± SEM. Data are representative of three to five different experiments. **p < 0.01, ***p < 0.005 compared with the wild type Treg-of-B (P) cells or responder T only group. ^#^*p* < 0.05, ^###^*p* < 0.005, compared with wild type or DMSO treated Treg-of-B (P).

We further confirmed the role of STAT6 on Treg-of-B (P) cells by culturing wild-type Peyer’s patch B cells with wild-type T cells in the presence of STAT6 inhibitor, AS, or STAT6 knockout (STAT6KO) T cells for three days. First, we assessed whether STAT1, STAT3 and STAT5 were affected in the absence of phosphorylated STAT6. After culture with Peyer’s patch B cells, a lack of phosphorylated STAT6 did not affect STAT1, STAT3 and STAT5 phosphorylation in STAT6KO T cells or AS treated T cells (**Figure 2A**). The lack of phosphorylated STAT6 attenuated the expression of LAG3 and the suppressive ability of Treg-of-B (P) cells (**Figures 2B, C**) but did not affect molecules that have been reported to mediate regulatory T cell function, including cytotoxic T- lymphocyte-associated antigen 4 (CTLA4), glucocorticoid-induced TNFR family-related gene (GITR), inducible T cell costimulator (ICOS), OX40, programmed death 1 (PD-1) and tumor necrosis factor receptor superfamily, member 1b (TNFRII) (**Supplementary Figure 1**). Moreover, Treg-of-B (P) cells lacking STAT6 secreted lower levels of IL-4 and IL-10, but similar level of IFNγ, compared with the wild-type group (**Figure 2D**). These data suggested that STAT6 played a major role in mediating the Treg-of-B (P) cell generation. Without STAT6, even normal phosphorylated STAT1, STAT3, and STAT5 would not help functional Treg-of-B (P) cell generation.

### B Cells Provide the Environment to Induce Phosphorylated STAT6 and LAG3 in Treg-of-B (P) Cells

**Figure 3:**
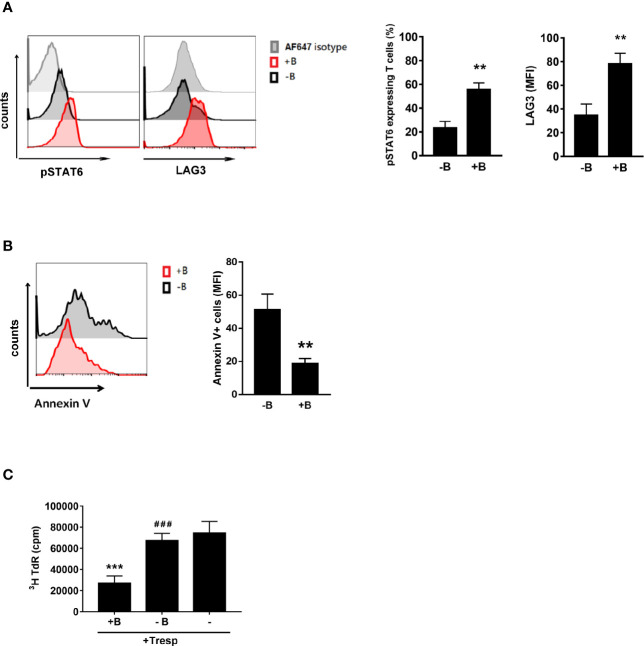
Peyer’s patch B cells provide the environment for Treg-of-B (P) cell generation with the phosphorylated STAT6 and LAG3 expression. To determine whether Peyer’s patch B cells are important for Treg-of-B (P) cells generation through STAT6-LAG3 pathway, CD25- CD4+ T cells were cultured with or without Peyer’s patch B cells in presence of anti-CD3 plus anti-CD28 1 µg/ml for 3 days. CD4+ cells were gated to represent Treg-of-B (P) cells in B-T cocultured. Phosphor-STAT6 and LAG3 **(A)** and the Annexin V **(B)** expressed by T cells or Treg-of-B (P) cells were analyzed by flow cytometry. **(C)** After three day cocultured, Peyer’s patch B cells were depleted and T cells were harvested to determine the suppressive function. Red line, T cells cultured with B cells (+B); black line, T cells cultured without B cells (-B); gray line, isotype control. Results are expressed as the mean ± SEM. Data are representative of four different experiments. **p < 0.01, ***p < 0.005, compared with the -B group or responder T cell group. ^###^*p* < 0.005, compared +B group.

We demonstrated that B cells could generate Treg cells (6–10, 21). Here, we wanted to investigate whether Peyer’s patch B cells are essential for inducing Treg-of-B (P) cells through STAT6 activation. First, we stimulated CD25- CD4+ T cells with anti-CD3 plus anti-CD28 antibodies in the presence or absence of Peyer’s patch B cells and found that phosphorylated STAT6 and LAG3 expression was decreased when B cells were lacking (**Figure 3A**). Without B cells, T cells could not be converted into Treg cells with impaired suppressive ability (**Figure 3C**). The existence of B cells was required for maintaining the viability of Treg-of-B (P) cells (**Figure 3B**). This result suggested that B cells could provide the factors to induce Treg-of-B (P) cell *via* STAT6 phosphorylation.

### STAT6-Deficient T Cells Could Not be Converted Into Treg Cells and Exerted No Therapeutic Effect in the Allergic Asthma Model

**Figure 4:**
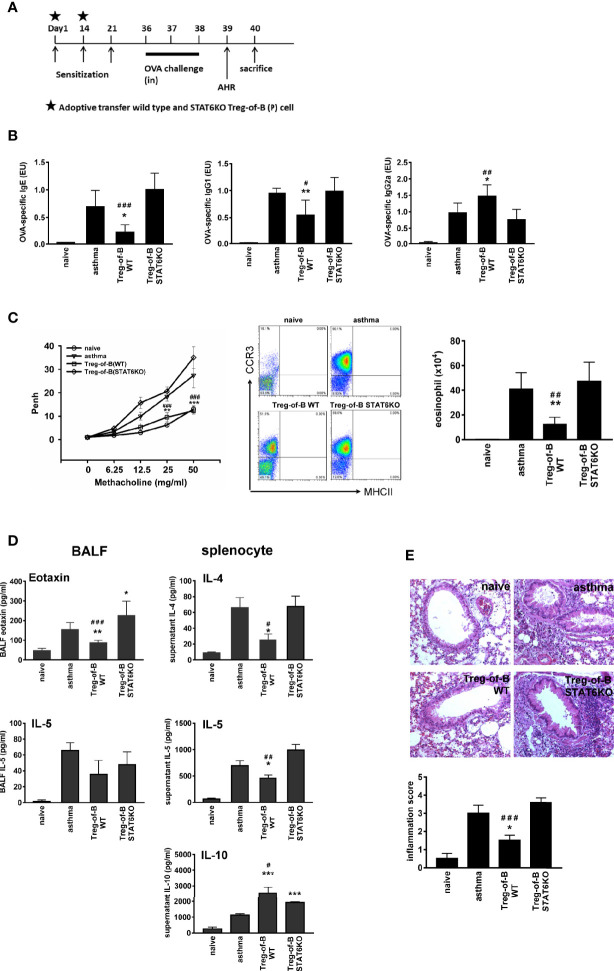
STAT6 deficient Treg-of-B (P) cells did not exert therapeutic effect on allergic asthma. **(A)** Sensitization protocol. Mice were primed on days 1, 14 and 21 and challenges were performed on days 36–38. The Wild type Treg-of-B (P) cells or STAT6 knockout Treg-of-B (P) cells were adoptively transferred into mice on day 1 and 14 (2.5 × 10^6^/mouse). Forty-eight h later, mice were sacrificed and parameters were analyzed. Airway hyperresponsiveness (AHR) was assessed one day prior sacrifice. **(B)** Serum was collected to measure the levels of OVA-specific IgE, IgG1 and IgG2a. The antibody concentrations in the standard serum were arbitrarily set to 1 ELISA unit (EU), where EU = (a sample – a blank)/(a positive – a blank). **(C)** The airway hyperresponsiveness following methacholine (MCh) challenge was performed and infiltrated eosinophils (MHCII- CCR3+) in bronchial alveolar lavage fluid (BALF) was evaluated. **(D)** The IL-4, 5, and 10 and eotaxin levels in BALF and in the splenocyte culture supernatants were determined by ELISA. **(E)** Histological examination. Pulmonary tissue section of different groups stained with H&E and inflammation score was determined as described in Materials and Methods. The results are expressed as the mean ± SEM. Data are representative of three different experiments. *p < 0.05, **p < 0.01, ***p < 0.005, compared with the asthma group. ^#^*p* < 0.05 ^##^*p* < 0.001, ^###^*p* < 0.005, compared Treg-of-B STAT6KO group.

The role of STAT6 in mediating Treg cell function is controversial. One study mentioned that STAT6 was essential for antigen-specific CD4+ CD25+ Foxp3+ Treg cell generation (30). However, another study suggested that constitutive activation of the IL-4 receptor, which is STAT6-dependent, inhibits the generation of Treg cells (31). Our data demonstrated that Treg-of-B (P) cell exerting suppressive function is STAT6-dependent. Next, we wanted to determine whether this phenomenon could be observed *in vivo*. As demonstrated in our previous studies (6, 21), adoptive transfer of Treg-of-B (P) cells could inhibit allergic asthmatic symptoms, including decreased amounts of OVA-specific IgE and IgG1, elevated OVA-specific IgG2a, reduced airway hyperresponsiveness, and infiltrated eosinophil in BALF, lower amounts of eotaxin in BALF and inflammation score in lung tissue (**Figures 4B–E**). Moreover, IL-4 and IL-5 secreted by splenocytes, which belongs to systemic Th2 response, were downregulated in wild type Treg-of-B (P) group (**Figure 4D**). In contrast, STAT6-deficient T cells activated by Peyer’s patch B cells did not exert the effects on OVA-induced asthma parameters (**Figure 4**). Th2 responses in the STAT6KO Treg-of-B (P) group, including OVA-specific IgE and IgG1, IL-5 produced by splenocytes, eotaxin in BALF, airway hyperresponsiveness and infiltrated eosinophilia in BALF, were all comparable with those in the asthma group. These results indicated that Peyer’s patch B cells induced Treg-of-B (P) cells in a STAT6-dependent manner.

### IL-4, Which Is Downstream but Not Inducer of STAT6 Phosphorylation, Maintained the Viability of Treg-of-B (P) Cells

**Figure 5:**
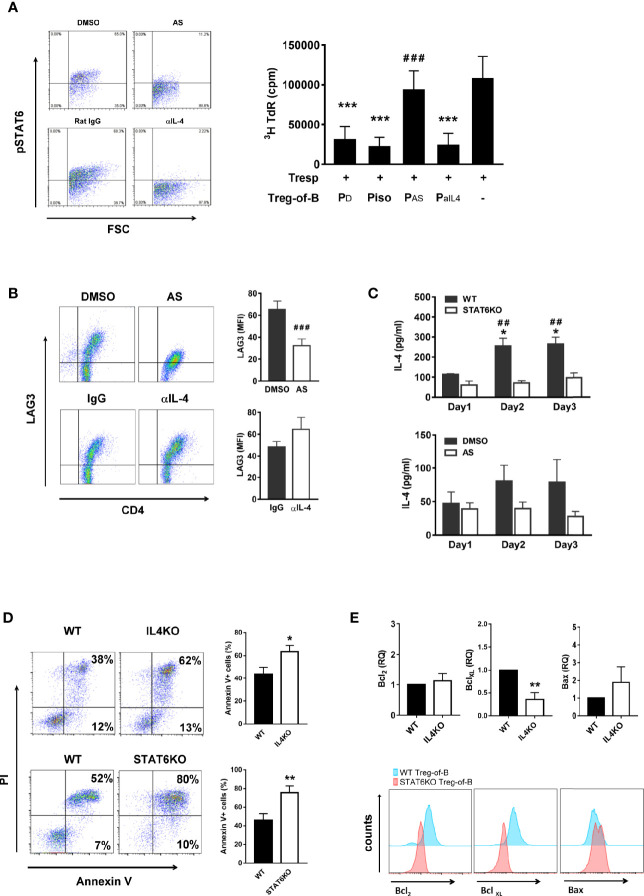
IL-4 was the downstream, not the inducer, of STAT6 phosphorylation in Treg-of-B (P) cells and responsible for Treg-of-B (P) cell viability maintenance. To determine whether IL-4 played the role in the generation of Treg-of-B (P) cells, CD4+CD25- T cells were cultured with anti-CD3, anti-CD28 antibodies and Peyer’s patch B cells in presence of DMSO, AS (50nM, STAT6 inhibitor), isotype IgG1 and anti-IL-4 (10 µg/ml) antibody. After B-T three-day cocultured, phosphorylated STAT6, LAG3, cell apoptosis and anti-apoptotic protein of Treg-of-B (P) were analyzed by flow cytometer. Supernatant of B-T coculture was collected at days 1 to 3 for IL-4 detection by ELISA. Gene expressions of anti-apoptotic and apoptotic protein were determined by real-time PCR. **(A)** phospho-STAT6 (left) expression and the suppressive ability of different treated groups of Treg-of-B (P) cell (right). **(B)** LAG3 expression of Treg-of-B (P) cells. **(C)** IL-4 production in B-T cell cocultured medium. Black bar: wild type group or DMSO group. White bar: STAT6KO group or AS group. **(D)** The apoptosis of wild type, IL-4 knockout (IL4KO) and STAT6 knockout (STAT6KO) Treg-of-B (P) cells. Apoptosis was evaluated by PI and Annexin V expression (early apoptosis: Annexin V+ PI-; late apoptosis: Annexin V+ PI+). Total apoptotic cells (Annexin V positive cells) were calculated for the bar graph. **(E)** The expressions of anti-apoptotic protein, Bcl2 and Bcl_XL_, and apoptotic protein, Bax, were quantified by real-time PCR (upper) and FACS analysis (lower) in IL4KO and STAT6KO Treg-of-B cells, respectively. RQ stands for the relative quantification of the PCR signal of IL4KO group compared to WT group. Results are expressed as the mean ± SEM. Data are representative of three to four different experiments. *p < 0.05, **p < 0.001, compared with WT group. ***p < 0.005, compared with the responder T cell group. ^##^*p* < 0.01, compared with STAT6KO group. ^###^*p* < 0.005 compared with DMSO group.

IL-4 is a well-known activator of STAT6 phosphorylation; moreover, phosphorylated STAT6 is upstream of IL-4 and promote IL-4 production (30). A previous study reported that IL-4 modulates Foxp3+CD4+ Treg cell function (24). Based on these findings, we hypothesized that IL-4 might participate in Treg-of-B (P) cell generation. The STAT6 inhibitor AS1517499 was used as a positive control to evaluate the effect of IL-4. First, we determined the effect of IL-4 on STAT6 phosphorylation. The results showed that phosphorylated STAT6 in T cells was diminished when T cells were cultured with Peyer’s patch B cells in the presence of anti-IL-4 antibody; however, neutralizing IL-4 did not affect Treg-of-B (P) cell generation with a significantly suppressive ability similar to isotype control groups (**Figure 5A**). In addition, the levels of LAG3 were not different in the IL-4 abolished group (**Figure 5B**). These results suggested that IL-4 did not affect Treg-of-B (P) cell induction. The amount of IL-4 in the Treg-of-B (P) cell generation system was also determined, and we found that IL-4 was increased in B-T cell cocultured medium from day 1 to day 3 in the wild-type or DMSO control group. In contrast, the amount of IL-4 remained unchanged when Treg-of-B (P) cells were generated in the absence of STAT6 phosphorylation (**Figure 5C**). This result suggested that IL-4 was regulated by phosphorylated STAT6 and was not involved in Treg-of-B (P) cell generation. IL-4 has been suggested to maintain cell survival; therefore, we examined the role of IL-4 in cell viability.

We determined the expression of the anti-apoptotic proteins, Bcl-2 and Bcl_XL_, and apoptotic protein, Bax in WT, IL-4 knockout (IL4KO) and STAT6 knockout Treg-of-B (P) cells. In the absence of IL-4 and STAT6, the apoptotic Treg-of-B cells were significantly increased (**Figure 5D**). The similar results were also shown in T cells treated with anti-IL-4 antibody (data not shown). Corresponding to the Annexin V results, Bax expression was increased in the IL-4KO and STAT6KO groups. Moreover, Bcl-2 and Bcl_XL_ were apparently expressed by wild type Treg-of-B cells, compared with IL-4KO and STAT6KO Treg-of-B cells (**Figure 5E**). These results indicated that B cells could stimulate T cells to phosphorylate STAT6, and then help T cells produce IL-4 to maintain cell viability.

## Discussion

Signal transducer and activator of transcription (STAT) family proteins are essential for T cell differentiation by transmitting cytokine signals to cells. The family contains several members, including STATs 1, 2, 3, 4, 5, and 6, and each STAT responds to different cytokines and modulates different T cell differentiation. For example, STAT1, which responds to IFNγ, is important for Th1 cells; STAT3, which responds to IL-6 and IL-21, is important for Th17 cells; STAT4, which responds to IL-12, is important for Th1 cells; STAT5, which responds to IL-2 and growth factor, is important for Treg cells. STAT6, which responds to IL-4, is important for Th2 cells (29, 32). There are some controversial points about STAT5 in Treg cells. CD4+ CD25+ regulatory T cells maintain self-tolerance *via* downregulation of the immune response (33). CD25 is IL-2Ra, which suggests that IL-2 signaling might be important in Treg cells. A previous study also demonstrated that STAT5-deficient mice suffered from autoimmune diseases, which emphasized the role of IL-2 in regulatory T cell induction (34). However, IL-2- and IL-2Ra- deficient Foxp3+ Treg cell numbers decreased, but the suppressive functions were similar to those of wild-type Treg cells (35, 36). This implies that IL-2/STAT5 could maintain the number of Treg cells but not regulate lineage development. The role of STAT6 in regulatory T cells has been previously discussed (30, 31, 37). STAT6 provides the secondary signal that is required for Foxp3+ Treg cell development (30). The results showed that in the absence of STAT6, thymus and peripheral Foxp3+ Treg cell numbers were decreased compared to those in wild-type mice, however, Treg cell function was not affected. The opposite role of STAT6 had been mentioned. The constitutive activation of STAT6 reprograms the Foxp3+ Treg cells into Th2-like cells in mice carrying the F709 mutation in the ITIM motif of the IL-4Ra chain (31). These Th2-like Foxp3+ Treg cells, which express Th2-related transcription factors and cytokines, exacerbate food allergy symptoms. All these studies were performed based on Foxp3+ Treg cells. Here, we demonstrated that STAT6 is an important molecule for inducing Foxp3 negative Treg-of-B (P) cells. Without STAT6, naïve T cells could not be converted into Treg-of-B (P) cells, given that the suppressive ability was abolished *in vitro* and *in vivo* (**Figures 1B**, **2C**, and **4**). Allergic responses in asthmatic mice, including airway hyperresponsiveness, OVA-specific IgE levels, eosinophil infiltration and Th2-related cytokines could not be alleviated by adoptive transfer of STAT6 deficient Treg-of-B (P) cells. These results demonstrated that STAT6 was critical for the function of Treg-of-B (P) cells.

Given that Peyer’s patch B cells are capable of inducing Treg-of-B (P) cells to alleviate the immune response (6, 21), we sought to determine whether B cells are associated with phosphorylated STAT6 and LAG3 expression. In accordance with our findings, CD4+ CD25- T cells activated by T cell receptor (TCR) signaling (anti-CD3/CD28) are insufficient to induce increased phosphorylated STAT6, LAG3 expression and suppressive function. This finding suggested that Peyer’s patch B cells could provide the environment to help Treg-of-B (P) cell generation (**Figure 3**). It has been demonstrated that IL-10 plays an important role in controlling asthma (38, 39). IL-10 exerted a direct effect on Th2 cells by regulating Th2 cell survival. Our data showed that STAT6KO Treg-of-B (P) cells secreted limited amounts of IL-10, compared to wild- type Treg-of-B (P) cells. In addition, LAG3 expression was decreased in STAT6KO Treg-of-B (P) cells. These findings prompted us to detect the function of STAT6KO Treg-of-B (P) cells *in vivo*. The lack of STAT6 led to impaired Treg-of-B (P) cell generation with no effect on alleviating asthma inflammatory parameters, whereas wild type Treg-of-B (P) cells exerted a regulatory function in a murine asthma model (**Figure 4**). The results showed that Peyer’s patch B cells provided the signal to phosphorylate STAT6 in T cells and this phosphorylated STAT6 was critical for converting naïve T cells into Treg-of-B (P) cells with regulatory function.

IL-4 was the first candidate when we assessed phosphorylated STAT6 activators. However, our data demonstrated that IL-4 was downstream of STAT6 phosphorylation and not the inducer, in the Treg-of-B (P) cell induction system. In addition, IL-4 did not affect the generation of Treg-of-B (P) cells (**Figures 5A–C**). Accordingly, we hypothesized that an unknown molecule produced by Peyer’s patch B cells activated T cells with STAT6 phosphorylation. Following phosphorylation of STAT6, IL-4 is generated and subsequently activates STAT6 with a positive feedback loop. Moreover, IL-4 could maintain Treg-of-B (P) cell viability to control the immune response. Therefore, it is important to identify the molecules provided by Peyer’s patch B cells that could trigger the serial reaction. Several molecules, including IL-13, GITR ligation, platelet-derived growth factor (PDGF), leptin, CD200 and vascular endothelial growth factor (VEGF), have been reported to activate STAT6 (40–44). We assayed these candidates that were detected in the B-T culture supernatant and found that none of them affected Treg-of-B (P) cell generation (data not shown). It is important to determine the molecule produced by Peyer’s patch B cells that would affect Treg-of-B (P) cell generation. In the future, more studies will be performed to identify potential molecules.

**Figure 6:**
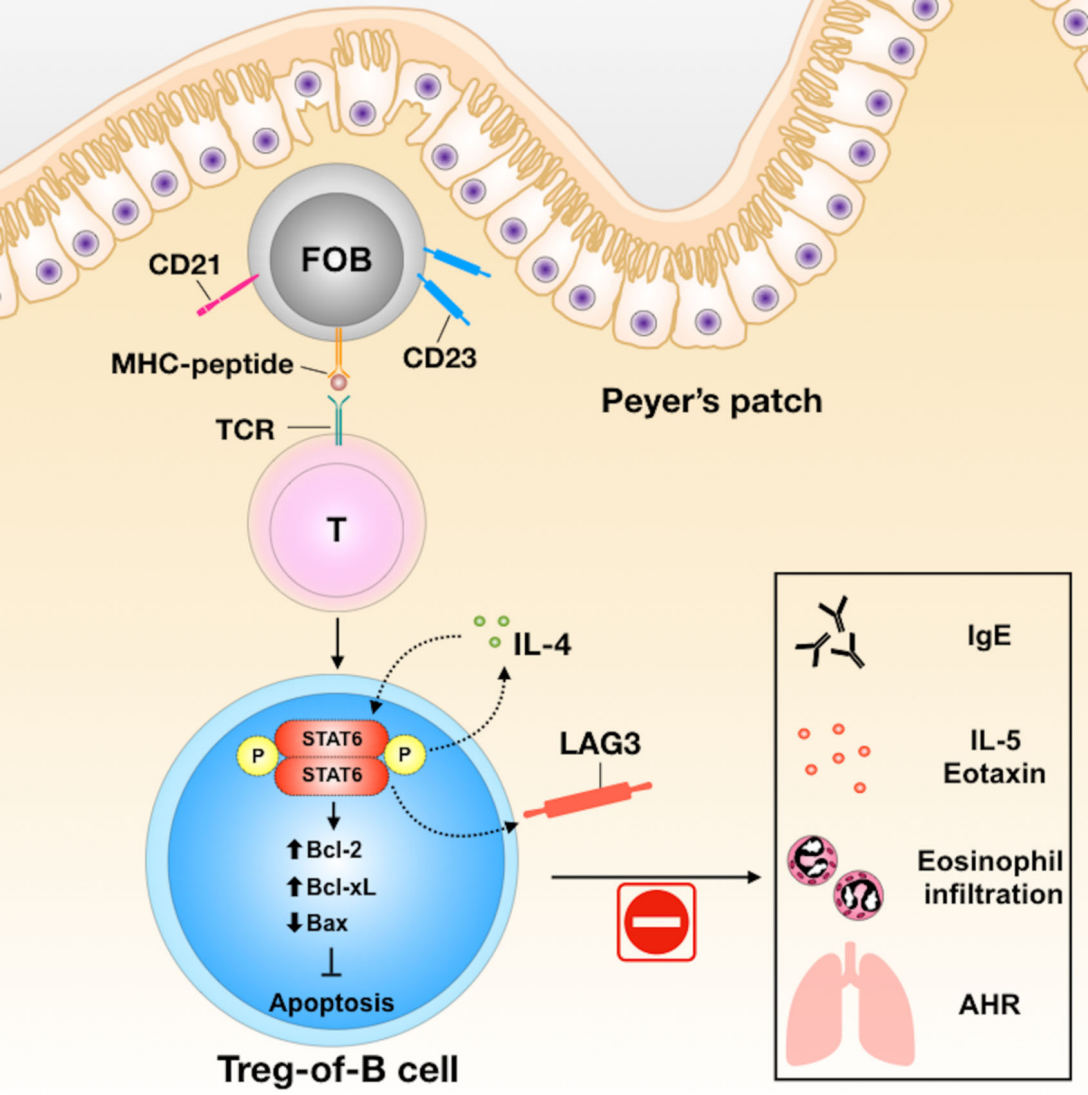
The summary of Treg-of-B (P) cell generation and their molecular mechanisms in alleviation of asthmatic inflammation. In the process of generation of Treg-of-B (P) cells, Peyer’s patch B cells would activate T cells with STAT6 phosphorylation. Phosphorylated STAT6 could trigger IL-4 production and then lead to positive feedback loop to increase the cell viability, *via* enhancing Bcl-2 and Bcl_XL_, and reducing Bax, and sustain the STAT6 phosphorylation. Phosphorylated STAT6 would regulate LAG3 expression, which mediated the suppressive ability of Treg-of-B (P) cells and alleviated murine model of airway inflammation.

In conclusion, Peyer’s patch B cells could induce Treg-of-B (P) cells through STAT6 phosphorylation and LAG3 expression. Treg-of-B (P) cells exert immunomodulatory activities both *in vitro* and *in vivo* (**Figure 6**). Further characterization of these Treg-of-B (P) cells might shed light on future therapeutic applications for immunological diseases.

## Data Availability Statement

The original contributions presented in the study are included in the article/**Supplementary Material**. Further inquiries can be directed to the corresponding author.

## Ethics Statement

The animal study was reviewed and approved by The Institutional Animal Care and Use Committee (IACUC) of the College of Medicine at National Taiwan University.

## Author Contributions

K-HC designed the study, performed most parts of experiments, analyzed the data, and drafted the manuscript. S-YL performed IL-4KO experiments. B-LC conceived the study and helped draft the manuscript. Both authors have read and approved the final manuscript. All authors contributed to the article and approved the submitted version.

## Funding

This study was supported by a grant, NHRI-EX108-10834SI, from the National Health Research Institute, Taiwan, Republic of China.

## Conflict of Interest

The authors declare that the research was conducted in the absence of any commercial or financial relationships that could be construed as a potential conflict of interest.
