# Supplementary material for: STAT6 Pathway Is Critical for the Induction and Function of Regulatory T Cells Induced by Mucosal B Cells
Source: Front Immunol. 2021 Jan 29;11:615868. doi: 10.3389/fimmu.2020.615868 (PMC7878545; doi:10.3389/fimmu.2020.615868)
Supplement: Supplementary file 1 [file Image_1.pdf]

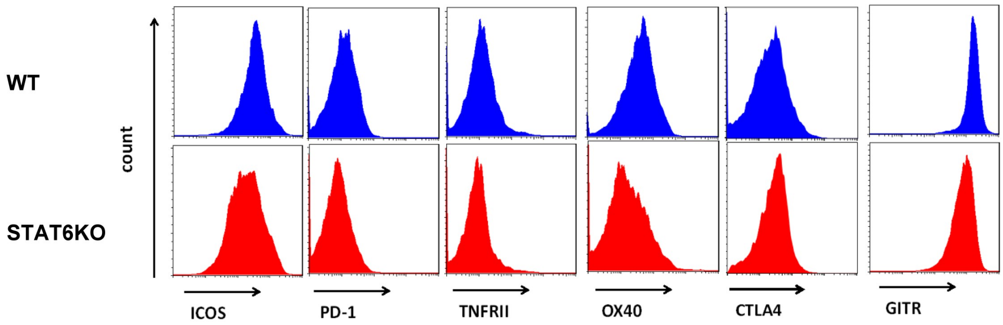

Supplementary Figure 1.

Supplementary Figure 1. Flow cytometry expression of ICOS, PD-1, TNERII, OX40, CTLA4 and GITR. Wild type or STAT6KO Treg-of-B (P) cells were harvested on day3 and applied to staining with fluorescent antibodies. Wild type, blue line. STAT6KO, red line. Data are representative of three different experiments.
